# The Nep1-like protein family of *Magnaporthe oryzae* is dispensable for the infection of rice plants

**DOI:** 10.1038/s41598-017-04430-0

**Published:** 2017-06-29

**Authors:** Ya-Li Fang, You-Liang Peng, Jun Fan

**Affiliations:** 1 Ministry of Agriculture Key Laboratory of Plant Pathology, China Agricultural University, Beijing, 100193 China; 2 State Key Laboratory of Agrobiotechnology, China Agricultural University, Beijing, 100193 China

## Abstract

The necrosis- and ethylene-inducing protein 1 (Nep1)-like proteins (NLPs) are a class of microbe-associated molecular patterns widely distributed across diverse groups of plant-associated microorganisms. In spite of the cytotoxic activity in dicot plants, the role of most NLPs in the virulence of plant pathogens is still largely unknown. We showed that the MoNLP family of rice blast fungus varied very little in amino acid sequence, transient expression of three *MoNLP*s induced cell death and the production of reactive oxygen species in *Nicotiana benthamiana*, and the expression of *MoNLPs* was induced during infection of susceptible rice plants. To further investigate the biological role of the *MoNLP* family, a marker-free gene replacement vector was developed and used to knock out the whole family in *Magnaporthe oryzae*. Results showed no significant difference in disease levels caused by wild type and the quadruple *ΔMoNLP* mutant strains. Likewise, the sporulation and radial growth of the two strains were similar under various unfavorable cultural conditions including malnutrition and abiotic stresses. These observations demonstrated that the MoNLP family is dispensable for the fungal tolerance to the tested adverse cultural conditions, and more importantly, for the virulence of blast fungus on susceptible rice plants.

## Introduction

Plants have developed a complex and multilayered immune system to detect and ward off invasions by diverse microbial pathogens. The first line of a plant’s surveillance system invokes the recognition of conserved molecules derived from diverse groups of microbes, also known as microbe-associated molecular patterns (MAMPs), by pattern recognition receptors (PRRs) distributed at the surface of plant cells^1^. An array of defense responses are subsequently induced including the production of reactive oxygen species, fortification of cell walls, upregulation of defense-related genes, and accumulation of antimicrobial compounds^2, 3^, which collectively confer plant resistance to adapted and non-adapted pathogens (also referred to as MAMP-triggered immunity, MTI).

A variety of microbial patterns or components have been described as MAMPs from bacteria, oomycetes, and fungi^4^. MAMPs from different microbial groups are normally distinct; however, a class of necrosis and ethylene-inducing protein 1 (Nep1)-like proteins (NLPs) have been reported as MAMPs from mostly plant-associated microbes across all three of the taxonomic groups^5–7^. The first described member of NLPs is Nep1, a 24-kDa protein that was purified from *Fusarium oxysporum* culture filtrates and capable of inducing necrosis and ethylene biosynthesis in dicot but not monocot plants^8^. The NLPs share a conserved NPP1 domain^7, 9^, and over 500 NLP-encoding genes have been identified based on sequence similarity analysis from microorganisms of diverse taxonomy and lifestyles^10^. Interestingly, the number of *NLP* family members can vary significantly among microorganisms. For instance, the wheat pathogen *Mycosphaerella graminicola* only has a single *NLP* gene in the genome^11^, whereas up to 33 copies of *NLP* genes have been identified from the genome of soybean pathogen *Phytophthora sojae* ^12^. In addition, studies have also demonstrated the versatile variation in sequence feature and necrosis-inducing activity across NLP family members^10, 13^. These observations indicate the existence of functional diversification of NLPs during complex biological processes in a broad range of microorganisms.

Apart from the ability to elicit MTI responses, the role of NLP in plant-microbe interaction is not fully understood, although the cytotoxicity of NLPs has been shown associated with the virulence of some pathogens on their dicot host plants^13–15^. Several observations indicate that NLPs may have roles independent of cytotoxicity: many NLPs are unable to cause plant cell death^12, 13^; multiple *NLPs* have been identified from obligate biotrophic pathogens^16^ as well as pathogens colonizing monocot host plants^11, 17, 18^. The impact of NLPs on the virulence of the pathogen under these non-cytotoxic conditions is still largely unknown. Motteram *et al*. (2009) have demonstrated that the only *NLP* gene of *M. graminicola* is dispensable for the fungal pathogen to cause disease on wheat plants but the role of a multi-membered *NLP* family in host colonization has not been reported.

Here, we describe our study on the potential biological role of NLP family in *Magnaporthe oryzae*, the causal agent of rice blast disease, which has four *MoNLPs* genes in the genome^7, 17^. Through a gain-of-function genetic screening, we initially identified a *MoNLP* gene that encoded an elicitor triggering typical immune responses in *Nicotiana benthamiana*. Subsequently, we investigated several aspects of the MoNLP family of the fungus including the amino acid sequence variation among lab strains and field isolates, expression profiles during infection, and the cytotoxicity of each member of the protein family. More importantly, we developed a pop-in/pop-out gene replacement vector and successfully deleted all four *MoNLP* genes from the genome of *M. oryzae*. The potential roles of MoNLPs in fungal virulence and tolerance to various adverse cultural conditions were further evaluated.

## Results

### Activation tagging identified an *MoNLP* gene from *M. oryzae* that elicited cell death when overexpressed in *N. benthamiana* plants

To search for proteinaceous elicitors from *M. oryzae* that can trigger immune responses in non-host plants, we modified an activation tagging vector previously used for generation of gain-of-function mutation in *Arabidopsis* ^19^, and obtained the binary vector pCB260-M (see Supplementary Fig. S1) used for construction of a genomic library of *M. oryzae* in *Agrobacterium tumefaciens*. We screened about 15,000 clones of the library by *Agrobacterium*-mediated transient expression and identified four clones that were capable of inducing either cell death or chlorosis in *N. benthamiana* plants (see Supplementary Table S1). Sequencing of the insert ends of G7, a cell death-inducing clone, revealed that the insert contained two annotated fungal genes *MGG_10531* and *MGG_10532* (see Supplementary Fig. S2A). To further determine which of these two genes was responsible for the cell death, restriction endonucleases that specifically disrupted the individual gene were used to truncate the G7 plasmid clone, and the transient expression assay showed that *MGG_10532*, which encodes the MoNLP4^17^, was both necessary and sufficient for the G7 induced cell death in *N. benthamiana* plants (see Supplementary Fig. S2B). Genes responsible for the elicitor activity of other three clones were subsequently identified with a similar approach, and they were predicted to encode two hypothetical proteins and a Ras-2 protein, respectively (see Supplementary Table S1). Among these candidate elicitors, MoNLP proteins have the predicted signal peptide for protein secretion, and the function of NLPs in the microorganism is still obscure; we therefore chose the MoNLP family for further investigation.

### The MoNLP family is highly conserved across strains of *M. oryzae*

Multiple studies have revealed that NLP family members can vary significantly in number among different microbial species (see Supplementary Table S2) although they share the highly conserved domain^7^, indicating the potential differentiation in NLP function associated with distinct microorganisms. Previous studies have identified four NLP-encoding genes in the blast fungus: *MGG_08454* (*MoNLP1*), *MGG_00401* (*MoNLP2*), *MGG_02332* (*MoNLP3*) and *MGG_10532* (*MoNLP4*)^7, 17^. We investigated the sequence variation of MoNLPs among three sequenced *M*. *oryzae* strains^20^ and a panel of field isolates collected from different geographic regions of China. The results showed that the MoNLP family rarely varied: MoNLP1, MoNLP2, and MoNLP4 were individually identical, and MoNLP3 shared 99.6 ~ 100% of identity across the 21 tested strains and isolates (see Supplementary Fig. S3), implying that MoNLPs may have important roles in the biology of *M*. *oryzae*.

### Expression of *MoNLPs* triggered oxidative burst and cell death in *N. benthamiana*

**Figure 1:**
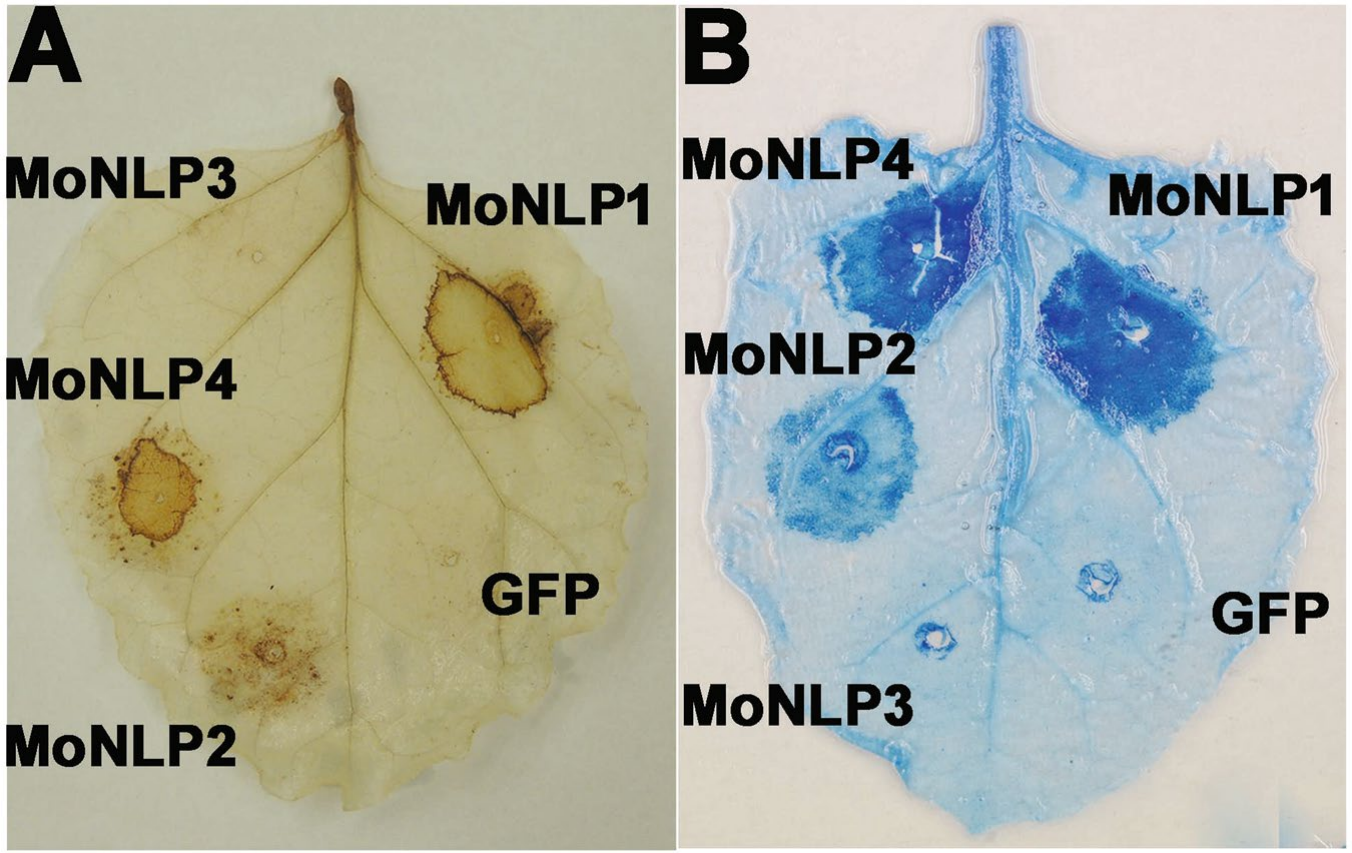
Transient expression of genes encoding necrosis and ethylene-inducing protein 1 (Nep1)-like protein (NLP) from *Magnaporthe oryzae* in *Nicotiana benthamiana*. MoNLP1, MoNLP2 and MoNLP4 trigger the accumulation of reactive oxygen species (**A**) and cell death (**B**), but not MoNLP3 and GFP control. Agrobacteria harboring individual *MoNLP* or *GFP* genes under the control of estrogen-inducible promoter were infiltrated into plant leaves. The estradiol solution (5 μM) was infiltrated into the inoculated area 24 hours post inoculation (hpi). Leaves were stained with DAB or trypan blue at 48 hpi.

Several NLPs have been shown to be able to induce cell death preferentially in dicotyledonous plants^9, 12, 14^. To investigate the MoNLP-induced cellular responses on *N. benthamiana* plants, cDNAs of individual *MoNLP* genes were cloned and engineered downstream of the inducible promoter of a modified vector of pER8^21^, and the resulting constructs were used for *Agrobacterium*-mediated transient expression assay. Tissue staining of *Agrobacterium-*infiltrated leaves showed the production of reactive oxygen species (ROS) and the cell death of plant cells at 24 hours after treatment with the chemical inducer for constructs expressing *MoNLP1*, *MoNLP2* and *MoNLP4*, but not for *MoNLP3* and the *GFP* control (Fig. 1), indicating that MoNLPs differed in cytotoxic activity on *N. benthamiana*.

### Members of the *MoNLP* family were induced during infection of rice plant

**Figure 2:**
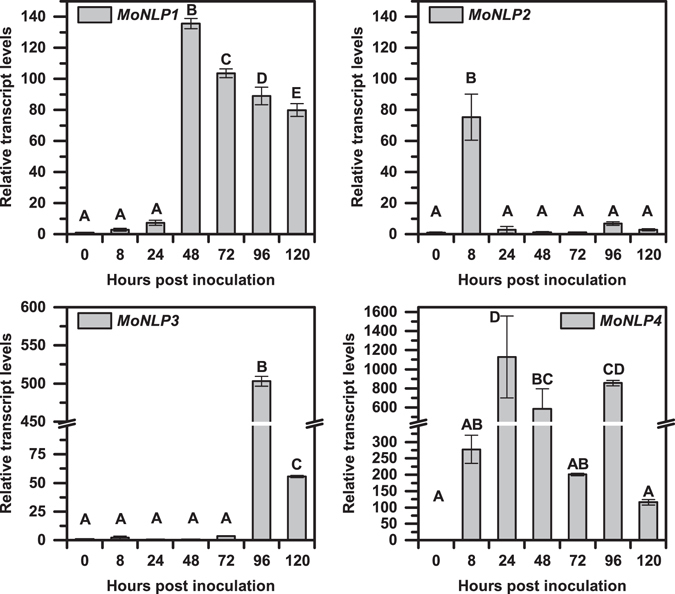
The relative transcript levels of *MoNLP* family are induced during infection of rice plants. Four-week-old rice plants were inoculated with *M. oryzae*, and samples were collected at indicated time points. The relative transcript levels of four *MoNLP* genes were determined by quantitative real-time polymerase chain reaction (q-RTPCR) using *Actin* and *40S ribosomal protein S3aE* of *M. oryzae* as reference genes and normalized against levels at 0 hpi. The conidial inoculum was used as the 0-hour sample. Data shown are means ± sd of three replicates. Statistical differences among the samples are labeled with different letters (ANOVA, P < 0.01). The experiment was repeated twice with similar results.

To investigate the potential role of MoNLPs during the interaction between *M. oryzae* and its host plant, we inoculated susceptible rice seedlings with wild type blast fungus strain P131, and analyzed levels of *MoNLP* transcripts throughout the infection process by quantitative real-time PCR. Results showed that all four members of the *MoNLP* family were up-regulated during the infection process but with seemingly distinct induction profiles. *MoNLP2* was strongly induced at 8 hours post inoculation (hpi) but subsided quickly afterward. Transcript levels of *MoNLP4* were significantly up-regulated at multiple time points in both early and late stage of the infection. The up-regulation of *MoNLP1* initiated from 48 hpi, when the symptom of chlorosis started to develop, whereas a significant transient induction of *MoNLP3* could be observed at a rather late stage of infection (96 hpi), when infected leaf tissue had already collapsed and fungal sporulation was visible on the abaxial surface of the inoculated leaf (Fig. 2). The diversified patterns of transcripts accumulation indicated that MoNLPs were likely involved in both biotrophic and necrotrophic phases of the infection.

### Knocking out the *MoNLP* family through pop-in/pop-out gene replacement

**Figure 3:**
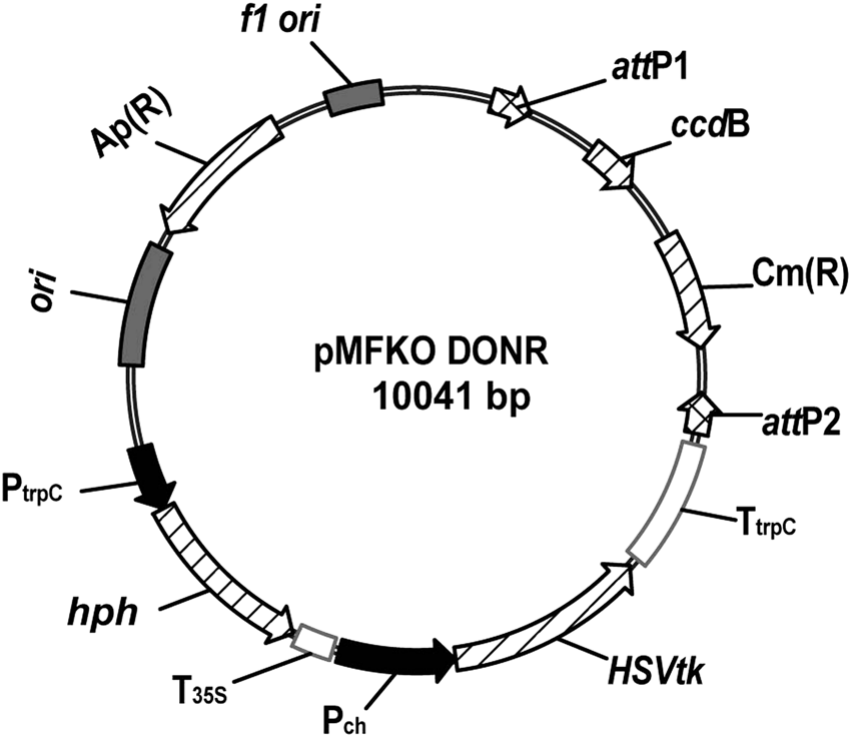
Schematic diagram of the vector pMFKO-DONR. *f1 ori*, phage f1 region; *att*P1 and *att*P2, Gateway sites; *ccd*B, coupled cell division B; Cm(R), chloramphenicol resistance gene; T_trpC_, terminator of *trpC* gene; *HSVtk*, thymidine kinase of herpes simplex virus; P_ch_, promoter region of *Cochliobolus heterostrophus* unknown protein presented in GenBank Accession Number M17304; T_35S_, CaMV 3’UTR (polyA signal); *hph*, hygromycin B phosphotransferase gene; P_trpC_, promoter region of *trpC* gene. *ori*, ColE1 origin of replication; Ap(R), ampicillin resistance gene.

**Figure 4:**
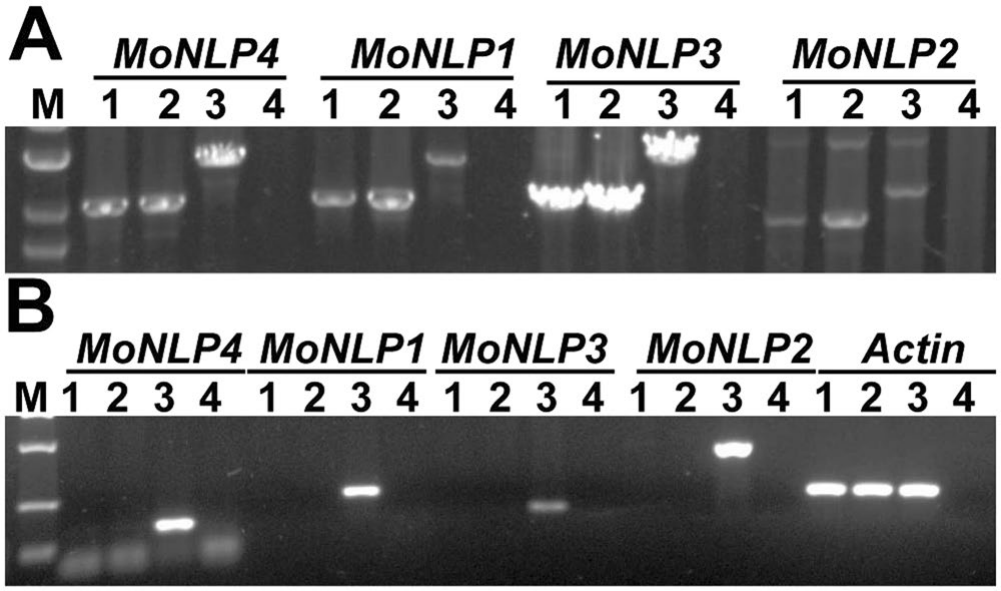
Validation of the loss-of *MoNLP* family in quadruple knockout strains by PCR. Genomic DNA of *MoNLP* knocked-out strains was amplified with specific primers annealing at sequences flanking the target regions subjected to homologous recombination (**A**) and with primers annealing at *MoNLP* coding regions (**B**). M indicates the molecular marker. Two independent knockout strains (1, 2), wild type *M. oryzae* (3), and water control (4) were assayed.

**Figure 5:**
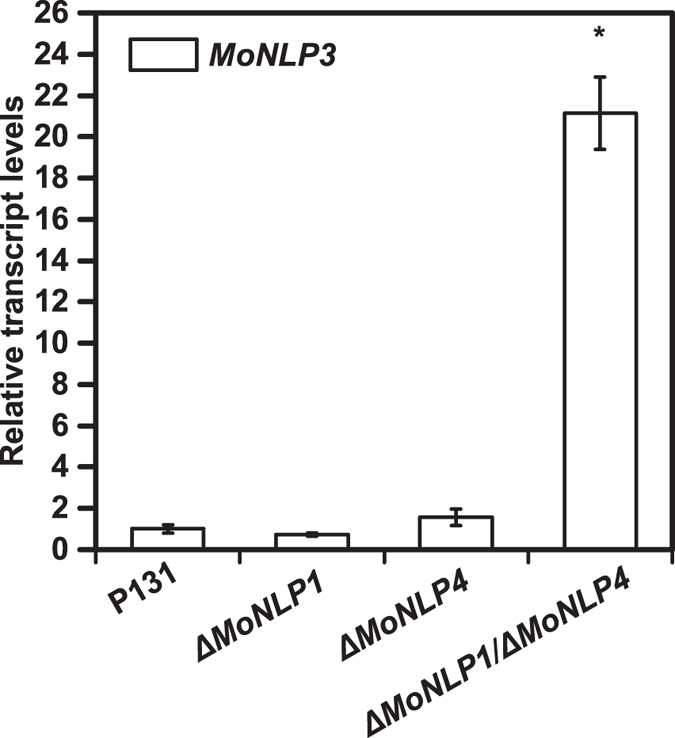
The expression of *MoNLP3* is significantly enhanced in *ΔMoNLP1/ΔMoNLP4* mutant during infection of rice. Leaves of 10-day-old rice seedlings were inoculated with wild type and mutants of *M. oryzae*, and samples were harvested at 96 hpi. Transcript levels of *MoNLP3* were determined by real-time PCR using *Actin* as the reference gene. Data shown are means ± sd of three replicates. Asterisk indicates a significant difference from P131 (Student’s t test, P < 0.05). The experiment was repeated at least twice with similar results.

To further elucidate whether the infection-associated activation of *MoNLPs* contributed to the virulence of blast fungus, we attempted to knock out all four members of the *MoNLP* family from the genome of *M. oryzae*. To perform the gene replacement, we firstly constructed a plasmid vector, pMFKO-DONR, which contained a hygromycin B resistance gene for positive selection, a conditional lethal *HSVtk* gene^22^ for negative selection, and the *att*P cassette of a pDONR vector (Gateway technology, Invitrogen) for cloning of DNA fragments required for *in situ* homologous recombination (Fig. 3). Subsequently, upstream and downstream DNA fragments of individual *MoNLP* coding regions were amplified and fused together by PCR and recombined with the vector by BP recombinase to produce pMFKO-NLP. These plasmids were used to sequentially delete all four *MoNLP* genes from the genome of P131 strain (Fig. 4). Interestingly, while we were constructing the quadruple mutant strain*ΔMoNLP*_1–4_, we found that knocking out either *MoNLP1* or *MoNLP4* had little impact on the expression of *MoNLP3* during infection but simultaneous disruption of both genes strongly enhanced transcript levels of *MoNLP3* at 96 hpi (Fig. 5). This synergistic effect on gene expression between *MoNLPs* indicated that certain levels of redundancy may exist between members of the *MoNLP* family during disease.

### The *MoNLP* family is dispensable for the infection of rice plants and the growth under various stress conditions

**Figure 6:**
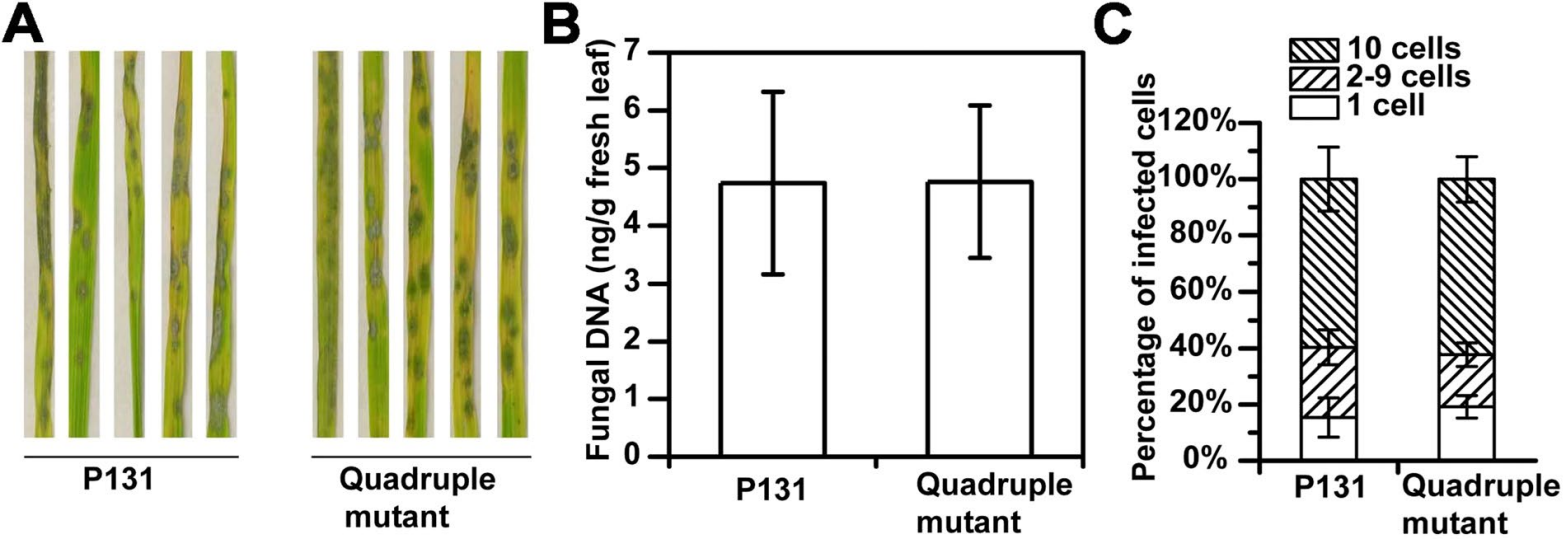
The *MoNLP* family is dispensable for virulence of *M. oryzae* on rice plants. (**A**) Disease symptoms caused by wild type and mutant fungal strains are similar on rice leaves. Ten-day-old susceptible rice seedlings (cv. LTH) were sprayed with conidial suspensions (1 × 10^5^ conidia ml^−1^) and incubated at room temperature with high relative humidity. Leaves were photographed at 5 days post inoculation. (**B**) The fungal growth of quadruple mutant is similar to that of wild type during infection. Samples were collected and weighted at 120 hpi, and DNA was extracted for qPCR assay of the fungal *Actin*. The fungal growth was quantitatively determined by the amount of fungal DNA in fresh leaves. (**C**) Percentages of types of infection sites classified by number of cells occupied by infectious hyphae on leaf sheath. The hollow space of sheaths (6.5 leaf stage) was injected with conidial suspension (5 × 10^3^ conidia ml^−1^). The infected tissues were stained with lactophenol cotton blue at 72 hpi. At least 40 infection sites were examined for each of the four plants used. Data shown in (**B**) are means ± sd (n = 4), and in (**C**) are means ± se (n = 4). Experiments were repeated at least twice with similar results.

Susceptible rice seedlings were spray-inoculated with the quadruple mutant and wild type P131 strains to investigate the potential role of *MoNLP* genes in fungal virulence. Five days after inoculation, similar levels of disease symptom in terms of the size and density of lesions were observed on plant leaves for both treatments (Fig. 6A). Likewise, the biomass of the quadruple mutant and wild type strains in the inoculated leaves hardly differed as indicated by the quantitative PCR analysis of the fungal DNA in diseased samples (Fig. 6B). We also inoculated detached rice leaves with conidial droplets and observed no significant difference in disease symptom and fungal biomass between quadruple mutants and wild type strains (see Supplementary Fig. S4). Moreover, we examined the growth of infectious hyphae around infection sites on the leaf sheath of rice plants. Penetration sites of single spores were classified into three categories based on the number of plant cells (1, 2–9 and > 10 cells) that the infectious hyphae had colonized. At 72 hpi, the percentages of each category of the infection sites were similar between wild type P131 and the quadruple mutant strain (Fig. 6C), indicating that deletion of the *MoNLP* gene family did not affect the fungal growth on leaf sheath as well. These results collectively demonstrated that the *MoNLP* gene family is not required for *M. oryzae* to infect the rice plant.

To determine whether the *MoNLP* family was required for the growth of *M. oryzae*, wild type and the quadruple mutant strains were grown on CM medium, 1.2% agar, Czapek-Dox medium, and nitrogen or carbon starvation medium. Results showed that the radial growth of the quadruple mutant was similar to that of wild type strain on these media (see Supplementary Fig. S5A). In addition, both strains produced similar levels of conidia when cultured on the oatmeal-tomato agar (OTA) medium (see Supplementary Fig. S5B). These findings suggested that the *MoNLP* family was not central to the growth and sporulation of *M. oryzae* under these conditions.

To examine the role of *MoNLP* family in the fungal response to diverse stress conditions, wild type and the quadruple mutant strains were grown on rich medium supplemented with SDS, congo red, 1.2 M sorbitol, and the iron chelator diethylenetriamine pentaacetic acid (DTPA), respectively. Radial growth of the quadruple mutant was found similar to that of the wild type strain (see Supplementary Fig. S6A), indicating that disruption of the *MoNLP* family did not compromise the integrity of fungal cell wall and membrane, and the tolerance to osmotic stress or iron limitation. Likewise, the growth of the two strains showed no difference under diverse pH (5.0 and 8.0) (see Supplementary Fig. S6A) or temperatures (15 °C and 32 °C) (see Supplementary Fig. S6B), suggesting that the gene family is not required for tolerance to these adverse conditions.

## Discussion

Current understanding of the biological role of NLPs during disease process is very limited, although genes encoding this class of proteins have been found in a wide range of microorganisms. Previous studies have shown that four genes encoding NLP exist in the genome of *M. oryzae* ^7, 17^. Our analysis revealed a remarkable conservation of MoNLPs among all the tested field isolates and lab strains (see Supplementary Fig. S3), implying that these genes may be essential for the survival of *M. oryzae* in nature. Interestingly, we observed that transcript levels of all four *MoNLPs* were induced in either or both of the biotrophic and necrotrophic phases during the infection of susceptible rice plants (Fig. 2). The up-regulation of *NLP* gene expression during infection has been described in other plant-pathogen interactions as well. In necrotrophic fungal pathogen *Botrytis cinerea*, the induction of *BcNep1* and *BcNep2* is at early and late stage of infection, respectively^23^. In addition, transcript levels of most of the expressed *PsNLPs* in *P. sojae* ^12, 24^ and two *VdNLPs* in *V. dahliae* ^13^ are highly induced at the late stage of infection; in contrast, the induction of four *HaNLPs* in the obligate biotroph oomycete *Hyaloperonospora arabidopsidis* occurs at the early stage of infection^16^. Notably, in hemibiotroph *M. graminicola*, the expression of the only *NLP* gene is prominently enhanced during a specific period near the end of the symptomless stage of the infection process^11^. These observations indicate that NLPs are naturally involved in the interactions between microbial pathogens and their host plants, but the complexity of gene induction profiles implies that these proteins may have diversified roles during infections.

NLPs are often regarded as virulence factors of pathogens on dicots due to the cytotoxic activity^25, 26^. For instance, ectopic expression of an *NLP* gene from *F. oxysporum* in *Colletotrichum coccdes*, a fungal pathogen used for biological control of weeds, strongly enhances the virulence of *C. coccdes* on the weed *Abutilon theophrasti* ^27^; silencing several *PcNLPs* in *P. capsici* leads to reduced virulence on pepper leaves^15^. However, the impact of some cytotoxic NLPs on pathogen virulence on dicots appears to be host dependent. For example, *VdNLP1* and *VdNLP2* are two genes encoding cytotoxic NLPs from *V. dahliae*, and disruption of either *VdNLP* compromises the fungal virulence on tomato and *Arabidopsis*, whereas, on *N. benthamiana* plants, the disruption of only *VdNLP1* but not *VdNLP2* affects the virulence^13^; the fungal virulence is not affected on cotton plants even when both genes were disrupted^28^. Likewise, the EccNip from *Erwinia carotovora* subsp. *carotovora* is required for full virulence of bacteria on potato tuber but not in potato stem or on other tested host plants^14^. Moreover, other studies show that deletion of a member of *NLP* family in *B. cinerea* or *F. oxysporum* does not impair the fungal virulence on tomato, *N. benthamiana* ^23^, or coca plants^29^, suggesting that NLPs may be dispensable for the virulence of pathogens. It is also possible that in these studies additional *NLP* genes may compensate the loss of a member of the family, which may mask the potential phenotype of mutant strains.

Gene family expansion is a common phenomenon in nature that leads to gene redundancy and functional diversification, which have also been reported for NLPs^12, 13, 16, 30^. In our study, the expression of *MoNLP3* was significantly upregulated at 96 hpi in the *ΔMoNLP1/ΔMoNLP4* double mutant compared to single knock-out or wild type strains (Fig. 5), implying that MoNLPs may be functionally complementary during infection of rice plants. To eliminate the potential redundancy between family members, we therefore, for the first time, disrupted all four *MoNLP* genes in the blast fungus, and results showed that the loss of entire multi-membered MoNLP family did not compromise the fungal virulence on rice (Fig. 6). This is unexpected especially considering the extensive upregulation of *MoNLPs’* expression during the infection process. However, it cannot be ruled out that other components or systems in *M. oryzae* may compensate the impact of the loss of MoNLP family on fungal virulence, and mask the phenotype of the quadruple mutant; alternatively, the enhanced *MoNLPs’* expression may be responsive to unknown stimuli generated during the infection process rather than boosting the fungal virulence *per se*. Previous studies also show that deletion of the only *MgNLP* from *M. graminicola* does not affect fungal virulence on wheat^11^. These results collectively demonstrate that the NLP family are not essential for fungal infection of monocot plants.

Results of previous research show that NLPs from monocot-colonizing pathogens do not trigger necrosis on monocots^11, 18^, *NLP* genes are activated in obligate biotrophic pathogens during infection^16^, and notably, insect pathogenic fungi, non-pathogens, and other animal-related fungi also carry *NLP* genes in their genomes^7, 10^. Hence, it is reasonable to assume that NLPs may have essential roles independent of phytotoxicity during certain fundamental biological processes. It has been reported that *VdNLP1* is required for the vegetative growth and production of conidiospore in *V. dahliae* ^13^, and several *VdNLP*s are induced at a later stage of cultivation in liquid Czapek-Dox medium or by supplementing the medium with cotton root^28^. However, vegetative growth and sporulation were not affected in the quadruple mutant of *M. oryzae* in this study (see Supplementary Fig. S4). Many NLPs have a signal peptide and are secreted outside of the cells^8, 9^, and evidence also shows that the expression of *SsNep2* in *S. sclerotiorum* is upregulated by the physical properties of the contact surface^31^. Thus, the rich variation in *NLP* expression profiles may reflect the complexity of environmental cues encountered by microbes during their life cycle. Further investigation of the nature of these cues may provide helpful clues for dissecting the biological roles of MoNLPs.

In this study, we developed the vector pMFKO-DONR to delete four *MoNLP* genes in *M. oryzae* via the pop-in/pop-out method. This marker-free approach is commonly used for homologous replacement of genes in bacteria^32^, and is especially convenient when multiple genes are targeted for deletion. The efficiency of homologous recombination depends on where the target gene is located in the genome, and it has been reported that the average rate of targeted gene replacement is only about 7% in *M. grisea* ^33^. Our results demonstrated that this approach works well in the blast fungus. Thus, the vector pMFKO-DONR and related experimental protocols may be useful for reverse genetic study in other haploid fungi where potential gene redundancy is involved as well.

## Materials and Methods

### Isolation of genomic DNA

For PCR screening of *in situ* transformants, the genomic DNA of *M. oryzae* was isolated from mycelia grown in liquid CM medium for 3 days according to Cenis^34^. To construct the genomic library, mycelia were harvested and ground in liquid nitrogen, and DNA was extracted with cetyltrimethylammonium bromide (CTAB) according to the protocols^35, 36^.

### Construction of *M. oryzae* genomic library for activation tagging

The binary vector pJFAT260 used for generation of activation tagging lines of *Arabidopsis* ^19^ was digested with *Spe*I and ligated to reverse the orientation of the TAIL-PCR anchor and 4 × 35 S enhancer repeats. The resulting plasmid, designated as pCB260-M, was digested with *Bam*HI to remove part of the T-DNA region unnecessary for transient expression assay. The *Bam*HI digested vector was ligated to size fractionated genomic DNA (about 4–8 kb) of *M. oryzae* derived from partial digestion with *Sau*3AI. The ligation products were transformed into *Agrobacterium* AGL1 strain by electroporation. Individual clones grown on agar plates supplemented with kanamycin (50 μg ml^−1^) were cultured overnight in 96-well plates at 28 °C, and stored in 40% glycerol at −80 °C for further study.

### Sequence similarity analyses

Amino acid sequences of MoNLP family were aligned by Clustal W, and the percentage of identity was calculated by DNASTAR software.

### ***Agrobacterium***-mediated expression on ***N. benthamiana***

*A. tumefaciens* clones were grown at 28 °C overnight in LB medium supplemented with kanamycin (50 μg ml^−1^) until OD_600_ reached 2.0. Bacterial cells were collected by centrifugation and resuspended in distilled water. The bacterial suspensions were adjusted to OD_600_ = 0.5 and infiltrated with a needleless syringe into the leaves of *N. benthamiana*.

### Histochemical staining

Trypan blue staining was performed as described^37^. For DAB staining, samples were incubated in the DAB staining solution (1 mg ml^−1^, pH 3.8) overnight, and destained 5–10 min subsequently in boiling ethanol^38^.

The inoculated sheath was fixed using formaldehyde (ethanol-formaldehyde-acetic acid, 80:3.5:5, by vol.) as described^39^. The plant material was stained with lactophenol cotton blue for 6 hours at room temperature^40^.

### Vector construction

To construct the vector pCBER DEST used for inducible expression of *MoNLP* genes, the *att*R cassette of a Destination vector compatible with the GATEWAY™ cloning technology was cloned into *Xho*I and *Spe*I sites of the target expression cassette of the pER8 vector^21^. This modified inducible transcription unit and the G10–90-XVE cassette of pER8 were amplified by PCR, and cloned into the *Hin*dIII and *Xba*I (blunted) sites, respectively, of pCB302^41^ to obtain pCBER DEST.

To construct pMFKO-DONR, the *att*P cassette of pDONR201 (Invitrogen, USA) was amplified by PCR and cloned into the pGEM-T Easy vector (Promega, USA) carrying the *hygromycin B phosphotransferase* gene. The resulting plasmid was linearized by PCR with primer pair pTGD For and pTGD Rev (see Supplementary Table S3), and further ligated with the negative selection marker *HSVtk* gene that was amplified from the plasmid pHH-5^42^ to obtain pMFKO-DONR (Fig. 3).

To disrupt *MGG_10531* in the G7 plasmid, *Sma*I and *Hin*dIII were used to digest the G7 plasmid, and the resulting fragment was blunted and self-ligated to obtain the plasmid clone Δ*MGG_10531*. Likewise, *Cla*I and *Pst*I were used to digest G7 plasmid to disrupt *MGG_10532*, and the resulting fragment was blunted and self-ligated to obtain the plasmid cloneΔ*MGG_10532*.

### Targeted disruption of four *MoNLP* genes

Genomic DNA fragments over 1 kb in length flanking the coding region of the targeted *MoNLP* gene were amplified and fused together by PCR with gene-specific primers bearing *att*B sites at 5′-ends (see Supplementary Table S3). This DNA product was introduced into the pMFKO-DONR by BP recombinase to produce the vector pMFKO-NLP, which was used for the PEG/CaCl_2_ mediated transformation of the protoplasts of blast fungus^43, 44^. The transformed protoplasts were selected on 1% top agar supplemented with 250 μg ml^−1^ hygromycin B, and the resulting transformants were further screened for *in situ* homologous recombination by PCR analysis. Colonies bearing targeted integrations were transferred to OTA medium containing 5 μM 5-fuoro-2′-deoxyuridine (F2dU) to enrich cells having lost the counter-selection marker *HSVtk* gene. Conidia were harvested and grown on CM medium supplemented with 100 μM F2dU, and resistant candidates were subjected to PCR analysis to obtain mutants with the deletion of targeted *MoNLP* gene. The primers used for knocking out *MoNLP* genes are listed in Supplementary Table S3.

### Gene expression analysis

Total RNA was extracted from about 100 mg inoculated leaves by Trizol (Generay), and cDNA was synthesized by M-MLV (Takara). Transcript levels of *MoNLP* family were determined by quantitative real-time RT-PCR (Takara). The genes encoding Actin and 40 S ribosomal protein S3aE of *M. oryzae* were used as reference genes. Each sample has three technical repeats. The experiments were repeated at least twice. Primer pairs used in this study were listed in Supplementary Table S3.

### Plate assay of fungal growth, sporulation and stress tolerance

Radial growth of fungal mycelia was determined by placing 5 μl conidial suspension (1 × 10^4^ conidia ml^−1^ in water) on the center of an agar plate supplemented with various ingredients^45^. The inoculated plates were incubated at 28 °C in darkness. The diameter of colonies was measured at 6 days. The media used for assays include the CM medium^46^, CM medium supplemented with 1.2 M sorbitol or 200 μg/ml congo red^47^, 1.2% agar, Czapek-Dox medium, nitrogen or carbon starvation medium^48^ and rice medium with pH 8.0 or 5.0^49^, 1/10 CM medium supplemented with 8 μM DTPA^50^.

For quantification of sporulation, *M. oryzae* strains were cultured on OTA for 12 days^51^. Each strain has three repeats. Four discs (φ5 mm) harvested about 1 cm from the edge of plate were put into 2 ml tube, and 0.5 ml sterile water was added. Then, each sample was shaken vigorously for 1 minute. The conidial suspension was counted by haemocytometer.

### Plant inoculation

Fresh conidia were harvested from OTA medium with sterile water containing 0.02% Tween-20 as described^51^. For virulence detection, about ten-day-old rice seedlings were spray inoculated with conidial suspension^51^. Four pots (about 20 seedlings in a pot) were treated with each strain. For microscopic observation, the hollow space of rice leaf sheath from 5-week-old plants was inoculated with conidial suspension^52, 53^. In order to examine the expression of *MoNLP* genes during infection of rice, the detached leaves of about 4-week-old rice were placed in plates with moist filter paper, and 20 μl conidial suspension (1 × 10^6^ conidia ml^−1^ or 5 × 10^4^ conidia ml^−1^) was dropped on the leaves. All pots and plates were placed in a moist plastic box at 25 °C for the first 24 hours in darkness, and then transferred to a chamber with a photoperiod of 16 hours under fluorescent light.

For fungal biomass assay, genomic DNA of infected leaves was isolated, and the amount of fungal DNA was quantified with qPCR as previously described^54^.

### Data Availability

The datasets generated during and/or analysed during the current study are available from the corresponding author on reasonable request.

## Electronic supplementary material

Supplementary Information
