# Supplementary material for: The Nep1-like protein family of Magnaporthe oryzae is dispensable for the infection of rice plants
Source: Sci Rep. 2017 Jun 29;7:4372. doi: 10.1038/s41598-017-04430-0 (PMC5491491; doi:10.1038/s41598-017-04430-0)
Supplement: Supplementary file 1 — Supplementary Information [file 41598_2017_4430_MOESM1_ESM.pdf]

# The Nep1-like protein family of *Magnaporthe oryzae* is dispensable for the infection of rice plants

**Ya-Li Fang[1], You-Liang Peng[1,2] and Jun Fan[1*]**

[1] Ministry of Agriculture Key Laboratory of Plant Pathology, China Agricultural University, Beijing 100193, China

[2]State Key Laboratory of Agrobiotechnology, China Agricultural University, Beijing 100193, China

[*] corresponding: jfan@cau.edu.cn

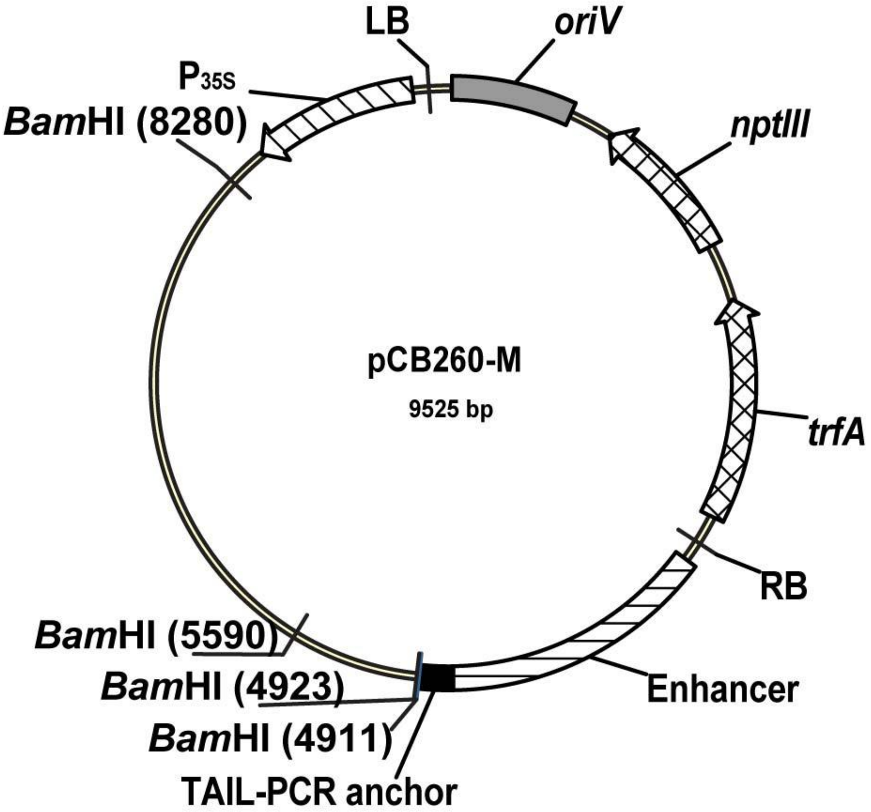


Fig. S1. Schematic diagram of pCB260-M. $P_{35S}$, 35S promoter of cauliflower mosaic virus; LB and RB, left and right border of T-DNA; *oriV*, replication origin from RK2; *nptIII*, neomycin phosphotransferase for kanamycin resistance; *trfA*, the replication protein gene of RK2; Enhancer, 4×35S enhancer repeats.

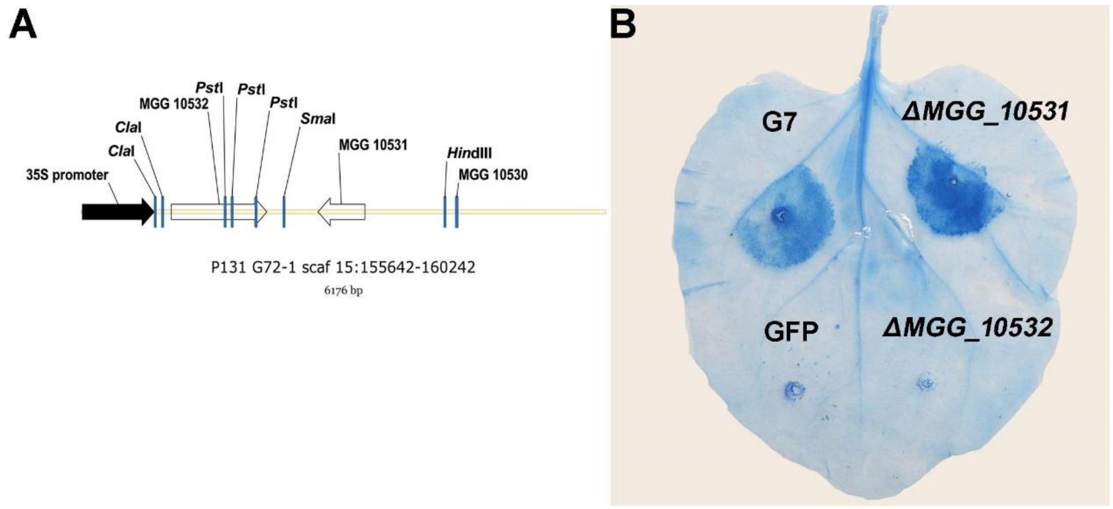


Fig. S2. *MGG_10532* of G7 clone triggers cell death by *Agro*-mediated transient expression in *Nicotiana benthamiana*. (A) Diagram of the genomic region from *Magnaporthe oryzae* in G7 clone. (B) Cell death-inducing activity of G7 clone is abolished in Δ*MGG_10532* but not in Δ*MGG_10531*clone. Leaves were stained with trypan blue at 72 hours post inoculation, and destained subsequently before the photograph was taken.

## MoNLP1

Percent Identity (upper triangle) / Divergence (lower triangle)

| | 1 | 2 | 3 | 4 | 5 | 6 | 7 | 8 | 9 | 10 | 11 | 12 | 13 | 14 | 15 | 16 | 17 | 18 | 19 | 20 | 21 | | |
|---|---|---|---|---|---|---|---|---|---|---|---|---|---|---|---|---|---|---|---|---|---|---|---|
| 1 | | 100.0 | 100.0 | 100.0 | 100.0 | 100.0 | 100.0 | 100.0 | 100.0 | 100.0 | 100.0 | 100.0 | 100.0 | 100.0 | 100.0 | 100.0 | 100.0 | 100.0 | 100.0 | 100.0 | 100.0 | 1 | P131 |
| 2 | 0.0 | | 100.0 | 100.0 | 100.0 | 100.0 | 100.0 | 100.0 | 100.0 | 100.0 | 100.0 | 100.0 | 100.0 | 100.0 | 100.0 | 100.0 | 100.0 | 100.0 | 100.0 | 100.0 | 100.0 | 2 | Y34 |
| 3 | 0.0 | 0.0 | | 100.0 | 100.0 | 100.0 | 100.0 | 100.0 | 100.0 | 100.0 | 100.0 | 100.0 | 100.0 | 100.0 | 100.0 | 100.0 | 100.0 | 100.0 | 100.0 | 100.0 | 100.0 | 3 | 70-15 |
| 4 | 0.0 | 0.0 | 0.0 | | 100.0 | 100.0 | 100.0 | 100.0 | 100.0 | 100.0 | 100.0 | 100.0 | 100.0 | 100.0 | 100.0 | 100.0 | 100.0 | 100.0 | 100.0 | 100.0 | 100.0 | 4 | isolate1 |
| 5 | 0.0 | 0.0 | 0.0 | 0.0 | | 100.0 | 100.0 | 100.0 | 100.0 | 100.0 | 100.0 | 100.0 | 100.0 | 100.0 | 100.0 | 100.0 | 100.0 | 100.0 | 100.0 | 100.0 | 100.0 | 5 | isolate2 |
| 6 | 0.0 | 0.0 | 0.0 | 0.0 | 0.0 | | 100.0 | 100.0 | 100.0 | 100.0 | 100.0 | 100.0 | 100.0 | 100.0 | 100.0 | 100.0 | 100.0 | 100.0 | 100.0 | 100.0 | 100.0 | 6 | isolate3 |
| 7 | 0.0 | 0.0 | 0.0 | 0.0 | 0.0 | 0.0 | | 100.0 | 100.0 | 100.0 | 100.0 | 100.0 | 100.0 | 100.0 | 100.0 | 100.0 | 100.0 | 100.0 | 100.0 | 100.0 | 100.0 | 7 | isolate4 |
| 8 | 0.0 | 0.0 | 0.0 | 0.0 | 0.0 | 0.0 | 0.0 | | 100.0 | 100.0 | 100.0 | 100.0 | 100.0 | 100.0 | 100.0 | 100.0 | 100.0 | 100.0 | 100.0 | 100.0 | 100.0 | 8 | isolate5 |
| 9 | 0.0 | 0.0 | 0.0 | 0.0 | 0.0 | 0.0 | 0.0 | 0.0 | | 100.0 | 100.0 | 100.0 | 100.0 | 100.0 | 100.0 | 100.0 | 100.0 | 100.0 | 100.0 | 100.0 | 100.0 | 9 | isolate6 |
| 10 | 0.0 | 0.0 | 0.0 | 0.0 | 0.0 | 0.0 | 0.0 | 0.0 | 0.0 | | 100.0 | 100.0 | 100.0 | 100.0 | 100.0 | 100.0 | 100.0 | 100.0 | 100.0 | 100.0 | 100.0 | 10 | isolate7 |
| 11 | 0.0 | 0.0 | 0.0 | 0.0 | 0.0 | 0.0 | 0.0 | 0.0 | 0.0 | 0.0 | | 100.0 | 100.0 | 100.0 | 100.0 | 100.0 | 100.0 | 100.0 | 100.0 | 100.0 | 100.0 | 11 | isolate8 |
| 12 | 0.0 | 0.0 | 0.0 | 0.0 | 0.0 | 0.0 | 0.0 | 0.0 | 0.0 | 0.0 | 0.0 | | 100.0 | 100.0 | 100.0 | 100.0 | 100.0 | 100.0 | 100.0 | 100.0 | 100.0 | 12 | isolate9 |
| 13 | 0.0 | 0.0 | 0.0 | 0.0 | 0.0 | 0.0 | 0.0 | 0.0 | 0.0 | 0.0 | 0.0 | 0.0 | | 100.0 | 100.0 | 100.0 | 100.0 | 100.0 | 100.0 | 100.0 | 100.0 | 13 | isolate10 |
| 14 | 0.0 | 0.0 | 0.0 | 0.0 | 0.0 | 0.0 | 0.0 | 0.0 | 0.0 | 0.0 | 0.0 | 0.0 | 0.0 | | 100.0 | 100.0 | 100.0 | 100.0 | 100.0 | 100.0 | 100.0 | 14 | isolate11 |
| 15 | 0.0 | 0.0 | 0.0 | 0.0 | 0.0 | 0.0 | 0.0 | 0.0 | 0.0 | 0.0 | 0.0 | 0.0 | 0.0 | 0.0 | | 100.0 | 100.0 | 100.0 | 100.0 | 100.0 | 100.0 | 15 | isolate12 |
| 16 | 0.0 | 0.0 | 0.0 | 0.0 | 0.0 | 0.0 | 0.0 | 0.0 | 0.0 | 0.0 | 0.0 | 0.0 | 0.0 | 0.0 | 0.0 | | 100.0 | 100.0 | 100.0 | 100.0 | 100.0 | 16 | isolate13 |
| 17 | 0.0 | 0.0 | 0.0 | 0.0 | 0.0 | 0.0 | 0.0 | 0.0 | 0.0 | 0.0 | 0.0 | 0.0 | 0.0 | 0.0 | 0.0 | 0.0 | | 100.0 | 100.0 | 100.0 | 100.0 | 17 | isolate14 |
| 18 | 0.0 | 0.0 | 0.0 | 0.0 | 0.0 | 0.0 | 0.0 | 0.0 | 0.0 | 0.0 | 0.0 | 0.0 | 0.0 | 0.0 | 0.0 | 0.0 | 0.0 | | 100.0 | 100.0 | 100.0 | 18 | isolate15 |
| 19 | 0.0 | 0.0 | 0.0 | 0.0 | 0.0 | 0.0 | 0.0 | 0.0 | 0.0 | 0.0 | 0.0 | 0.0 | 0.0 | 0.0 | 0.0 | 0.0 | 0.0 | 0.0 | | 100.0 | 100.0 | 19 | isolate16 |
| 20 | 0.0 | 0.0 | 0.0 | 0.0 | 0.0 | 0.0 | 0.0 | 0.0 | 0.0 | 0.0 | 0.0 | 0.0 | 0.0 | 0.0 | 0.0 | 0.0 | 0.0 | 0.0 | 0.0 | | 100.0 | 20 | isolate17 |
| 21 | 0.0 | 0.0 | 0.0 | 0.0 | 0.0 | 0.0 | 0.0 | 0.0 | 0.0 | 0.0 | 0.0 | 0.0 | 0.0 | 0.0 | 0.0 | 0.0 | 0.0 | 0.0 | 0.0 | 0.0 | | 21 | isolate18 |
| | 1 | 2 | 3 | 4 | 5 | 6 | 7 | 8 | 9 | 10 | 11 | 12 | 13 | 14 | 15 | 16 | 17 | 18 | 19 | 20 | 21 | | |

## MoNLP2

Percent Identity (upper triangle) / Divergence (lower triangle)

| | 1 | 2 | 3 | 4 | 5 | 6 | 7 | 8 | 9 | 10 | 11 | 12 | 13 | 14 | 15 | 16 | 17 | 18 | 19 | 20 | 21 | | |
|---|---|---|---|---|---|---|---|---|---|---|---|---|---|---|---|---|---|---|---|---|---|---|---|
| 1 | | 100.0 | 100.0 | 100.0 | 100.0 | 100.0 | 100.0 | 100.0 | 100.0 | 100.0 | 100.0 | 100.0 | 100.0 | 100.0 | 100.0 | 100.0 | 100.0 | 100.0 | 100.0 | 100.0 | 100.0 | 1 | P131 |
| 2 | 0.0 | | 100.0 | 100.0 | 100.0 | 100.0 | 100.0 | 100.0 | 100.0 | 100.0 | 100.0 | 100.0 | 100.0 | 100.0 | 100.0 | 100.0 | 100.0 | 100.0 | 100.0 | 100.0 | 100.0 | 2 | Y34 |
| 3 | 0.0 | 0.0 | | 100.0 | 100.0 | 100.0 | 100.0 | 100.0 | 100.0 | 100.0 | 100.0 | 100.0 | 100.0 | 100.0 | 100.0 | 100.0 | 100.0 | 100.0 | 100.0 | 100.0 | 100.0 | 3 | 70-15 |
| 4 | 0.0 | 0.0 | 0.0 | | 100.0 | 100.0 | 100.0 | 100.0 | 100.0 | 100.0 | 99.6 | 100.0 | 100.0 | 100.0 | 100.0 | 100.0 | 100.0 | 100.0 | 100.0 | 100.0 | 100.0 | 4 | isolate1 |
| 5 | 0.0 | 0.0 | 0.0 | 0.0 | | 100.0 | 100.0 | 100.0 | 100.0 | 100.0 | 100.0 | 100.0 | 100.0 | 100.0 | 100.0 | 100.0 | 100.0 | 100.0 | 100.0 | 100.0 | 100.0 | 5 | isolate2 |
| 6 | 0.0 | 0.0 | 0.0 | 0.0 | 0.0 | | 100.0 | 100.0 | 100.0 | 100.0 | 100.0 | 100.0 | 100.0 | 100.0 | 100.0 | 100.0 | 100.0 | 100.0 | 100.0 | 100.0 | 100.0 | 6 | isolate3 |
| 7 | 0.0 | 0.0 | 0.0 | 0.0 | 0.0 | 0.0 | | 100.0 | 100.0 | 100.0 | 100.0 | 100.0 | 100.0 | 100.0 | 100.0 | 100.0 | 100.0 | 100.0 | 100.0 | 100.0 | 100.0 | 7 | isolate4 |
| 8 | 0.0 | 0.0 | 0.0 | 0.0 | 0.0 | 0.0 | 0.0 | | 100.0 | 100.0 | 100.0 | 100.0 | 100.0 | 100.0 | 100.0 | 100.0 | 100.0 | 100.0 | 100.0 | 100.0 | 100.0 | 8 | isolate5 |
| 9 | 0.0 | 0.0 | 0.0 | 0.0 | 0.0 | 0.0 | 0.0 | 0.0 | | 100.0 | 100.0 | 100.0 | 100.0 | 100.0 | 100.0 | 100.0 | 100.0 | 100.0 | 100.0 | 100.0 | 100.0 | 9 | isolate6 |
| 10 | 0.0 | 0.0 | 0.0 | 0.0 | 0.0 | 0.0 | 0.0 | 0.0 | 0.0 | | 100.0 | 100.0 | 100.0 | 100.0 | 100.0 | 100.0 | 100.0 | 100.0 | 100.0 | 100.0 | 100.0 | 10 | isolate7 |
| 11 | 0.0 | 0.0 | 0.0 | 0.0 | 0.0 | 0.0 | 0.0 | 0.0 | 0.0 | 0.0 | | 100.0 | 100.0 | 100.0 | 100.0 | 100.0 | 100.0 | 100.0 | 100.0 | 100.0 | 100.0 | 11 | isolate8 |
| 12 | 0.0 | 0.0 | 0.0 | 0.0 | 0.0 | 0.0 | 0.0 | 0.0 | 0.0 | 0.0 | 0.0 | | 100.0 | 100.0 | 100.0 | 100.0 | 100.0 | 100.0 | 100.0 | 100.0 | 100.0 | 12 | isolate9 |
| 13 | 0.0 | 0.0 | 0.0 | 0.0 | 0.0 | 0.0 | 0.0 | 0.0 | 0.0 | 0.0 | 0.0 | 0.0 | | 100.0 | 100.0 | 100.0 | 100.0 | 100.0 | 100.0 | 100.0 | 100.0 | 13 | isolate10 |
| 14 | 0.0 | 0.0 | 0.0 | 0.0 | 0.0 | 0.0 | 0.0 | 0.0 | 0.0 | 0.0 | 0.0 | 0.0 | 0.0 | | 100.0 | 100.0 | 100.0 | 100.0 | 100.0 | 100.0 | 100.0 | 14 | isolate11 |
| 15 | 0.0 | 0.0 | 0.0 | 0.0 | 0.0 | 0.0 | 0.0 | 0.0 | 0.0 | 0.0 | 0.0 | 0.0 | 0.0 | 0.0 | | 100.0 | 100.0 | 100.0 | 100.0 | 100.0 | 100.0 | 15 | isolate12 |
| 16 | 0.0 | 0.0 | 0.0 | 0.0 | 0.0 | 0.0 | 0.0 | 0.0 | 0.0 | 0.0 | 0.0 | 0.0 | 0.0 | 0.0 | 0.0 | | 100.0 | 100.0 | 100.0 | 100.0 | 100.0 | 16 | isolate13 |
| 17 | 0.0 | 0.0 | 0.0 | 0.0 | 0.0 | 0.0 | 0.0 | 0.0 | 0.0 | 0.0 | 0.0 | 0.0 | 0.0 | 0.0 | 0.0 | 0.0 | | 100.0 | 100.0 | 100.0 | 100.0 | 17 | isolate14 |
| 18 | 0.0 | 0.0 | 0.0 | 0.0 | 0.0 | 0.0 | 0.0 | 0.0 | 0.0 | 0.0 | 0.0 | 0.0 | 0.0 | 0.0 | 0.0 | 0.0 | 0.0 | | 100.0 | 100.0 | 100.0 | 18 | isolate15 |
| 19 | 0.0 | 0.0 | 0.0 | 0.0 | 0.0 | 0.0 | 0.0 | 0.0 | 0.0 | 0.0 | 0.0 | 0.0 | 0.0 | 0.0 | 0.0 | 0.0 | 0.0 | 0.0 | | 100.0 | 100.0 | 19 | isolate16 |
| 20 | 0.0 | 0.0 | 0.0 | 0.0 | 0.0 | 0.0 | 0.0 | 0.0 | 0.0 | 0.0 | 0.0 | 0.0 | 0.0 | 0.0 | 0.0 | 0.0 | 0.0 | 0.0 | 0.0 | | 100.0 | 20 | isolate17 |
| 21 | 0.0 | 0.0 | 0.0 | 0.0 | 0.0 | 0.0 | 0.0 | 0.0 | 0.0 | 0.0 | 0.0 | 0.0 | 0.0 | 0.0 | 0.0 | 0.0 | 0.0 | 0.0 | 0.0 | 0.0 | | 21 | isolate18 |
| | 1 | 2 | 3 | 4 | 5 | 6 | 7 | 8 | 9 | 10 | 11 | 12 | 13 | 14 | 15 | 16 | 17 | 18 | 19 | 20 | 21 | | |

## MoNLP3

Percent Identity (upper triangle) / Divergence (lower triangle)

| | 1 | 2 | 3 | 4 | 5 | 6 | 7 | 8 | 9 | 10 | 11 | 12 | 13 | 14 | 15 | 16 | 17 | 18 | 19 | 20 | 21 | | |
|---|---|---|---|---|---|---|---|---|---|---|---|---|---|---|---|---|---|---|---|---|---|---|---|
| 1 | | 100.0 | 100.0 | 100.0 | 100.0 | 100.0 | 100.0 | 100.0 | 100.0 | 100.0 | 100.0 | 100.0 | 100.0 | 100.0 | 100.0 | 100.0 | 99.6 | 100.0 | 99.6 | 99.6 | 99.6 | 1 | P131 |
| 2 | 0.0 | | 100.0 | 100.0 | 100.0 | 100.0 | 100.0 | 100.0 | 100.0 | 100.0 | 100.0 | 100.0 | 100.0 | 100.0 | 100.0 | 100.0 | 99.6 | 100.0 | 99.6 | 99.6 | 99.6 | 2 | Y34 |
| 3 | 0.0 | 0.0 | | 100.0 | 100.0 | 100.0 | 100.0 | 100.0 | 100.0 | 100.0 | 100.0 | 100.0 | 100.0 | 100.0 | 100.0 | 100.0 | 99.6 | 100.0 | 99.6 | 99.6 | 99.6 | 3 | 70-15 |
| 4 | 0.0 | 0.0 | 0.0 | | 100.0 | 100.0 | 100.0 | 100.0 | 100.0 | 100.0 | 100.0 | 100.0 | 100.0 | 100.0 | 100.0 | 100.0 | 99.6 | 100.0 | 99.6 | 99.6 | 99.6 | 4 | isolate1 |
| 5 | 0.0 | 0.0 | 0.0 | 0.0 | | 100.0 | 100.0 | 100.0 | 100.0 | 100.0 | 100.0 | 100.0 | 100.0 | 100.0 | 100.0 | 100.0 | 99.6 | 100.0 | 99.6 | 99.6 | 99.6 | 5 | isolate2 |
| 6 | 0.0 | 0.0 | 0.0 | 0.0 | 0.0 | | 100.0 | 100.0 | 100.0 | 100.0 | 100.0 | 100.0 | 100.0 | 100.0 | 100.0 | 100.0 | 99.6 | 100.0 | 99.6 | 99.6 | 99.6 | 6 | isolate3 |
| 7 | 0.0 | 0.0 | 0.0 | 0.0 | 0.0 | 0.0 | | 100.0 | 100.0 | 100.0 | 100.0 | 100.0 | 100.0 | 100.0 | 100.0 | 100.0 | 99.6 | 100.0 | 99.6 | 99.6 | 99.6 | 7 | isolate4 |
| 8 | 0.0 | 0.0 | 0.0 | 0.0 | 0.0 | 0.0 | 0.0 | | 100.0 | 100.0 | 100.0 | 100.0 | 100.0 | 100.0 | 100.0 | 100.0 | 99.6 | 100.0 | 99.6 | 99.6 | 99.6 | 8 | isolate5 |
| 9 | 0.0 | 0.0 | 0.0 | 0.0 | 0.0 | 0.0 | 0.0 | 0.0 | | 100.0 | 100.0 | 100.0 | 100.0 | 100.0 | 100.0 | 100.0 | 99.6 | 100.0 | 99.6 | 99.6 | 99.6 | 9 | isolate6 |
| 10 | 0.0 | 0.0 | 0.0 | 0.0 | 0.0 | 0.0 | 0.0 | 0.0 | 0.0 | | 100.0 | 100.0 | 100.0 | 100.0 | 100.0 | 100.0 | 99.6 | 100.0 | 99.6 | 99.6 | 99.6 | 10 | isolate7 |
| 11 | 0.0 | 0.0 | 0.0 | 0.0 | 0.0 | 0.0 | 0.0 | 0.0 | 0.0 | 0.0 | | 100.0 | 100.0 | 100.0 | 100.0 | 100.0 | 99.6 | 100.0 | 99.6 | 99.6 | 99.6 | 11 | isolate8 |
| 12 | 0.0 | 0.0 | 0.0 | 0.0 | 0.0 | 0.0 | 0.0 | 0.0 | 0.0 | 0.0 | 0.0 | | 100.0 | 100.0 | 100.0 | 100.0 | 99.6 | 100.0 | 99.6 | 99.6 | 99.6 | 12 | isolate9 |
| 13 | 0.0 | 0.0 | 0.0 | 0.0 | 0.0 | 0.0 | 0.0 | 0.0 | 0.0 | 0.0 | 0.0 | 0.0 | | 100.0 | 100.0 | 100.0 | 99.6 | 100.0 | 99.6 | 99.6 | 99.6 | 13 | isolate10 |
| 14 | 0.0 | 0.0 | 0.0 | 0.0 | 0.0 | 0.0 | 0.0 | 0.0 | 0.0 | 0.0 | 0.0 | 0.0 | 0.0 | | 100.0 | 100.0 | 99.6 | 100.0 | 99.6 | 99.6 | 99.6 | 14 | isolate11 |
| 15 | 0.0 | 0.0 | 0.0 | 0.0 | 0.0 | 0.0 | 0.0 | 0.0 | 0.0 | 0.0 | 0.0 | 0.0 | 0.0 | 0.0 | | 100.0 | 99.6 | 100.0 | 99.6 | 99.6 | 99.6 | 15 | isolate12 |
| 16 | 0.0 | 0.0 | 0.0 | 0.0 | 0.0 | 0.0 | 0.0 | 0.0 | 0.0 | 0.0 | 0.0 | 0.0 | 0.0 | 0.0 | 0.0 | | 99.6 | 100.0 | 99.6 | 99.6 | 99.6 | 16 | isolate13 |
| 17 | 0.4 | 0.4 | 0.4 | 0.4 | 0.4 | 0.4 | 0.4 | 0.4 | 0.4 | 0.4 | 0.4 | 0.4 | 0.4 | 0.4 | 0.4 | 0.4 | | 99.6 | 100.0 | 100.0 | 100.0 | 17 | isolate14 |
| 18 | 0.0 | 0.0 | 0.0 | 0.0 | 0.0 | 0.0 | 0.0 | 0.0 | 0.0 | 0.0 | 0.0 | 0.0 | 0.0 | 0.0 | 0.0 | 0.0 | 0.4 | | 99.6 | 99.6 | 99.6 | 18 | isolate15 |
| 19 | 0.4 | 0.4 | 0.4 | 0.4 | 0.4 | 0.4 | 0.4 | 0.4 | 0.4 | 0.4 | 0.4 | 0.4 | 0.4 | 0.4 | 0.4 | 0.4 | 0.0 | 0.4 | | 100.0 | 100.0 | 19 | isolate16 |
| 20 | 0.4 | 0.4 | 0.4 | 0.4 | 0.4 | 0.4 | 0.4 | 0.4 | 0.4 | 0.4 | 0.4 | 0.4 | 0.4 | 0.4 | 0.4 | 0.4 | 0.0 | 0.4 | 0.0 | | 100.0 | 20 | isolate17 |
| 21 | 0.4 | 0.4 | 0.4 | 0.4 | 0.4 | 0.4 | 0.4 | 0.4 | 0.4 | 0.4 | 0.4 | 0.4 | 0.4 | 0.4 | 0.4 | 0.4 | 0.0 | 0.4 | 0.0 | 0.0 | | 21 | isolate18 |
| | 1 | 2 | 3 | 4 | 5 | 6 | 7 | 8 | 9 | 10 | 11 | 12 | 13 | 14 | 15 | 16 | 17 | 18 | 19 | 20 | 21 | | |

## MoNLP4

Percent Identity (upper triangle) / Divergence (lower triangle)

| | 1 | 2 | 3 | 4 | 5 | 6 | 7 | 8 | 9 | 10 | 11 | 12 | 13 | 14 | 15 | 16 | 17 | 18 | 19 | 20 | 21 | | |
|---|---|---|---|---|---|---|---|---|---|---|---|---|---|---|---|---|---|---|---|---|---|---|---|
| 1 | | 100.0 | 100.0 | 100.0 | 100.0 | 100.0 | 100.0 | 100.0 | 100.0 | 100.0 | 100.0 | 100.0 | 100.0 | 100.0 | 100.0 | 100.0 | 100.0 | 100.0 | 100.0 | 100.0 | 100.0 | 1 | P131 |
| 2 | 0.0 | | 100.0 | 100.0 | 100.0 | 100.0 | 100.0 | 100.0 | 100.0 | 100.0 | 100.0 | 100.0 | 100.0 | 100.0 | 100.0 | 100.0 | 100.0 | 100.0 | 100.0 | 100.0 | 100.0 | 2 | Y34 |
| 3 | 0.0 | 0.0 | | 100.0 | 100.0 | 100.0 | 100.0 | 100.0 | 100.0 | 100.0 | 100.0 | 100.0 | 100.0 | 100.0 | 100.0 | 100.0 | 100.0 | 100.0 | 100.0 | 100.0 | 100.0 | 3 | 70-15 |
| 4 | 0.0 | 0.0 | 0.0 | | 100.0 | 100.0 | 100.0 | 100.0 | 100.0 | 100.0 | 100.0 | 100.0 | 100.0 | 100.0 | 100.0 | 100.0 | 100.0 | 100.0 | 100.0 | 100.0 | 100.0 | 4 | isolate1 |
| 5 | 0.0 | 0.0 | 0.0 | 0.0 | | 100.0 | 100.0 | 100.0 | 100.0 | 100.0 | 100.0 | 100.0 | 100.0 | 100.0 | 100.0 | 100.0 | 100.0 | 100.0 | 100.0 | 100.0 | 100.0 | 5 | isolate2 |
| 6 | 0.0 | 0.0 | 0.0 | 0.0 | 0.0 | | 100.0 | 100.0 | 100.0 | 100.0 | 100.0 | 100.0 | 100.0 | 100.0 | 100.0 | 100.0 | 100.0 | 100.0 | 100.0 | 100.0 | 100.0 | 6 | isolate3 |
| 7 | 0.0 | 0.0 | 0.0 | 0.0 | 0.0 | 0.0 | | 100.0 | 100.0 | 100.0 | 100.0 | 100.0 | 100.0 | 100.0 | 100.0 | 100.0 | 100.0 | 100.0 | 100.0 | 100.0 | 100.0 | 7 | isolate4 |
| 8 | 0.0 | 0.0 | 0.0 | 0.0 | 0.0 | 0.0 | 0.0 | | 100.0 | 100.0 | 100.0 | 100.0 | 100.0 | 100.0 | 100.0 | 100.0 | 100.0 | 100.0 | 100.0 | 100.0 | 100.0 | 8 | isolate5 |
| 9 | 0.0 | 0.0 | 0.0 | 0.0 | 0.0 | 0.0 | 0.0 | 0.0 | | 100.0 | 100.0 | 100.0 | 100.0 | 100.0 | 100.0 | 100.0 | 100.0 | 100.0 | 100.0 | 100.0 | 100.0 | 9 | isolate6 |
| 10 | 0.0 | 0.0 | 0.0 | 0.0 | 0.0 | 0.0 | 0.0 | 0.0 | 0.0 | | 100.0 | 100.0 | 100.0 | 100.0 | 100.0 | 100.0 | 100.0 | 100.0 | 100.0 | 100.0 | 100.0 | 10 | isolate7 |
| 11 | 0.0 | 0.0 | 0.0 | 0.0 | 0.0 | 0.0 | 0.0 | 0.0 | 0.0 | 0.0 | | 100.0 | 100.0 | 100.0 | 100.0 | 100.0 | 100.0 | 100.0 | 100.0 | 100.0 | 100.0 | 11 | isolate8 |
| 12 | 0.0 | 0.0 | 0.0 | 0.0 | 0.0 | 0.0 | 0.0 | 0.0 | 0.0 | 0.0 | 0.0 | | 100.0 | 100.0 | 100.0 | 100.0 | 100.0 | 100.0 | 100.0 | 100.0 | 100.0 | 12 | isolate9 |
| 13 | 0.0 | 0.0 | 0.0 | 0.0 | 0.0 | 0.0 | 0.0 | 0.0 | 0.0 | 0.0 | 0.0 | 0.0 | | 100.0 | 100.0 | 100.0 | 100.0 | 100.0 | 100.0 | 100.0 | 100.0 | 13 | isolate10 |
| 14 | 0.0 | 0.0 | 0.0 | 0.0 | 0.0 | 0.0 | 0.0 | 0.0 | 0.0 | 0.0 | 0.0 | 0.0 | 0.0 | | 100.0 | 100.0 | 100.0 | 100.0 | 100.0 | 100.0 | 100.0 | 14 | isolate11 |
| 15 | 0.0 | 0.0 | 0.0 | 0.0 | 0.0 | 0.0 | 0.0 | 0.0 | 0.0 | 0.0 | 0.0 | 0.0 | 0.0 | 0.0 | | 100.0 | 100.0 | 100.0 | 100.0 | 100.0 | 100.0 | 15 | isolate12 |
| 16 | 0.0 | 0.0 | 0.0 | 0.0 | 0.0 | 0.0 | 0.0 | 0.0 | 0.0 | 0.0 | 0.0 | 0.0 | 0.0 | 0.0 | 0.0 | | 100.0 | 100.0 | 100.0 | 100.0 | 100.0 | 16 | isolate13 |
| 17 | 0.0 | 0.0 | 0.0 | 0.0 | 0.0 | 0.0 | 0.0 | 0.0 | 0.0 | 0.0 | 0.0 | 0.0 | 0.0 | 0.0 | 0.0 | 0.0 | | 100.0 | 100.0 | 100.0 | 100.0 | 17 | isolate14 |
| 18 | 0.0 | 0.0 | 0.0 | 0.0 | 0.0 | 0.0 | 0.0 | 0.0 | 0.0 | 0.0 | 0.0 | 0.0 | 0.0 | 0.0 | 0.0 | 0.0 | 0.0 | | 100.0 | 100.0 | 100.0 | 18 | isolate15 |
| 19 | 0.0 | 0.0 | 0.0 | 0.0 | 0.0 | 0.0 | 0.0 | 0.0 | 0.0 | 0.0 | 0.0 | 0.0 | 0.0 | 0.0 | 0.0 | 0.0 | 0.0 | 0.0 | | 100.0 | 100.0 | 19 | isolate16 |
| 20 | 0.0 | 0.0 | 0.0 | 0.0 | 0.0 | 0.0 | 0.0 | 0.0 | 0.0 | 0.0 | 0.0 | 0.0 | 0.0 | 0.0 | 0.0 | 0.0 | 0.0 | 0.0 | 0.0 | | 100.0 | 20 | isolate17 |
| 21 | 0.0 | 0.0 | 0.0 | 0.0 | 0.0 | 0.0 | 0.0 | 0.0 | 0.0 | 0.0 | 0.0 | 0.0 | 0.0 | 0.0 | 0.0 | 0.0 | 0.0 | 0.0 | 0.0 | 0.0 | | 21 | isolate18 |
| | 1 | 2 | 3 | 4 | 5 | 6 | 7 | 8 | 9 | 10 | 11 | 12 | 13 | 14 | 15 | 16 | 17 | 18 | 19 | 20 | 21 | | |

Fig. S3. The four necrosis- and ethylene-inducing protein (Nep1)-like proteins (NLP) from *M. oryzae* are conserved among lab strains and field isolates. The amino acid sequences of twenty-one isolates collected from Heilongjiang, Hunan, Liaoning, Guangzhou and Hubei province were analyzed using Cluster W method. The percentage of amino acid sequence identity and divergence was analyzed using DNA Star software.

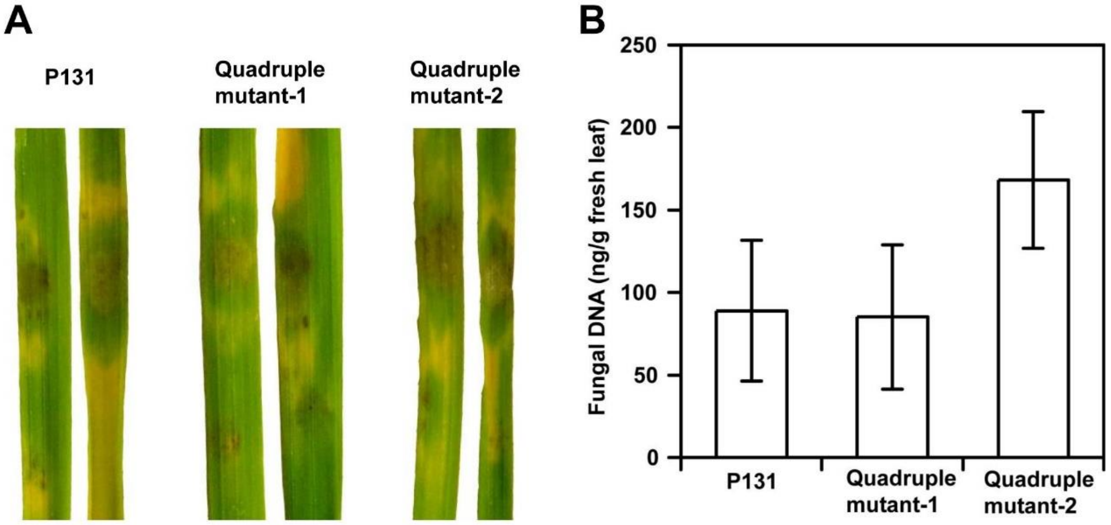


Fig. S4. The *MoNLP* family is dispensable for virulence of *M. oryzae* on rice plants. (A) Disease symptoms caused by wild type and two mutant fungal strains are similar on rice leaves. Susceptible rice seedlings (cv. LTH) were inoculated with 5 μl conidial suspensions ($2.5\times10^5$ conidia $ml^{-1}$) and incubated in moist plates at room temperature. Leaves were photographed at 3 days post inoculation. (B) The fungal growth of two independent quadruple mutants is similar to that of wild type during infection. Samples were collected and weighted at 72 hpi, and DNA was extracted for qPCR assay of the fungal *Actin*. The fungal growth was quantitatively determined by the amount of fungal DNA in fresh leaves. Data shown in (B) are means±sd (n=3). No significant difference was detected by statistical analysis (Student's t test, $P<0.05$)

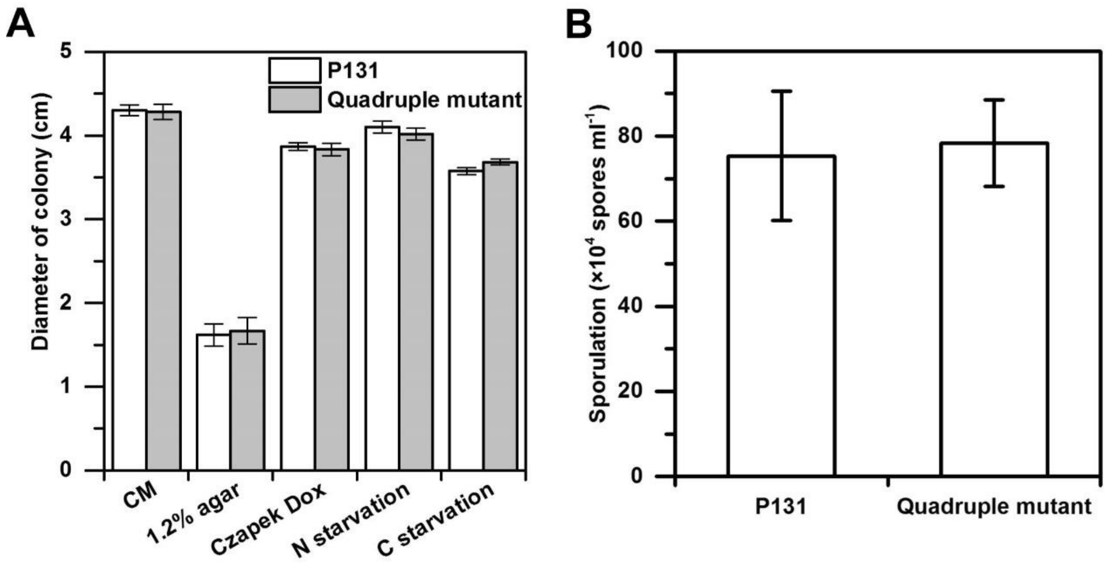


Fig. S5. Deletion of the *MoNLP* family does not affect the growth and sporulation of *M. oryzae*. (A) The colony diameters of wild type and quadruple mutant were measured after 6 days of incubation on CM medium, 1.2% agar, Czapek-Dox medium, and N or C starvation medium at 28°C. (B) A comparison of levels of conidia production by P131 and quadruple mutant. Data shown in (A) and (B) are means±sd (n=3). No significant difference was detected by statistical analysis (Student's t test, $P<0.05$)

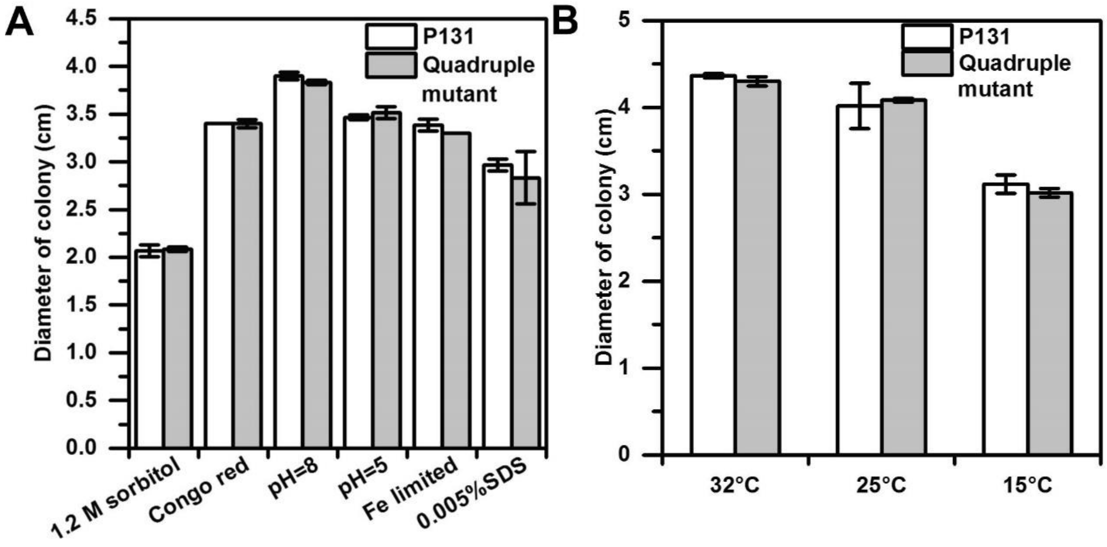


Fig. S6. Deletion of the *MoNLP* family does not affect the radial growth of *M. oryzae* under various adverse conditions. (A) The assays were performed by placing 5 μl conidial suspension ($1\times10^4$ conidian $ml^{-1}$) on the center of CM plates supplemented with 1.2 M sorbitol, SDS (0.005%) or congo red (200 μg/ml), 1/10 CM supplemented with DTPA (8 μM), and rice medium with pH 8.0 and 5.0. The inoculated plates were incubated at 28°C for 6 days before measuring the colony diameter. (B) The comparison of radial growth of wild type and mutant strains on PDA plates. The colony diameter was measured 7 days except for 12 days at 15°C after inoculation. Data shown in A and B are means±sd (n=3). No significant difference is detected between P131 and mutant strain (Student's t test, $P<0.05$).

Table S1 The candidate elicitor genes were identified by screening the genomic library of *M. oryzae*

| Candidate clones | Genes | Proteins | No. of family member | Signal peptide [a] | Phenotype |
|---|---|---|---|---|---|
| G7 | MGG_10532 | NEP1-like | 4 | Yes | Cell death |
| AD8 | MGG_04098 | Hypothetical protein1 | 1 | No | Cell death |
| G4L12 | MGG_14010 | Hypothetical protein 2 | 1 | No | Chlorosis |
| F6-H8 | MGG_09499 | Ras-2 | 2[b] | No | Chlorosis |

[a]Signal peptide was predicted by SignalP 4.0.

[b]The identity of amino acid sequence is over 50%.

Table S2 Number of NLP genes from diverse microbial species.

| Species | Number of *NLP* genes |
| --- | --- |
| *Mycosphaerella graminicola*[1] | 1 |
| *Erwinia carotovora subsp. carotovora,*[2] | 1 |
| *Moniliophthora perniciosa*[3] | 2 |
| *Botrytis elliptica*[4] | 2 |
| *B. cinerea*[5] | 2 |
| *Sclerotinia sclerotiorum*[6] | 2 |
| *Magnaporthe oryzae*[7] | 4 |
| *Verticillium dahliae*[8] | 8-9 |
| *Hyaloperonospora arabidopsidis*[9] | 12 |
| *Phytophthora sojae*[10] | 33 |
| *P. capsici*[11] | 60* |

* Potential NLPs based on genome sequencing

Table S3 Primers used for deletion of *MoNLP* family, construction of plasmid, and qRT-PCR assays

| Primer name | Primer Sequences (5'-3') | Remark |
|---|---|---|
| Mo10532 up For | **AAAAAGCAGGCT**CGAACATGATTAACAATTCCGGAG | MoNLP4 knock out vector construction |
| Mo10532 up Rev | ACATAACCACCCACTACATTGTGAATTTTGTGATGGGT | |
| Mo10532 down For | CACAAAATTCACAATGTAGTGGGTGGTTATGTGGTT | |
| Mo10532 down Rev | **AGAAAGCTGGGT**TACCTAGTTATTTCCCCTCC | |
| Mo10532 up left For | CAGGGTCTCCCATGCCAAGATT | Validation of MGG_10532 deletion |
| Mo10532 down right Rev | TCATCTTCCTCACTGCCGCCCT | |
| Mo08454 up For | **AAAAAGCAGGCT**CGATTGGCGATCTTTTGGATAC | MoNLP1 knock out vector construction |
| Mo08454 up Rev | AAGCCAACAAAGCTCACATCTTTTCTTTTTTCCTGAGGG | |
| Mo08454 down For | GAAAAAAGAAAAGATGTGAGCTTTGTTGGCTTTTTGG | |
| Mo08454 down Rev | **AGAAAGCTGGGT**AAAAGGACGGACTTGTTCATGCT | |
| Mo08454 up left For | CCTCCTTCTACATGCACCACTCCA | Validation of MGG_08454 deletion |
| Mo08454 down right Rev | CGCAGAGTGAATTCTGTGAGGACA | |
| Mo00401 up For-2 | **AAAAAGCAGGCT**TGATCTTGTAACTGTGTGGT | MoNLP2 knock out vector construction |
| Mo00401 up Rev-2 | AAAGACGGCGACGTGCCATGGTGTAGGATTAAAATG | |
| Mo00401 down For-2 | TTAATCCTACACCATGGCACGTCGCCGTCTTTGTCG | |
| Mo00401 down Rev-2 | **AGAAAGCTGGGT**CCCCTAAAACGACCCGAGATATTCG | |
| Mo00401 up left for-3 | ACCCTTACAAATCGCATGTCTTGT | Validation of MGG_00401 deletion |
| Mo00401 down right Rev | GCCGTTTTATAGCCCGATATCC | |
| Mo02332 up For-2 | AAAAAGCAGGCTAGAAATGGTTCTGACGGTGCATGT | MoNLP3 knock out vector construction |
| Mo02332 up Rev | CAGGACAAACCAATTACATGTTTCTCGGCTCGATCA | |
| Mo02332 down For | CGAGCCGAGAAACATGTAATTGGTTTGTCCTGTTGTT | |
| Mo02332 down Rev-2 | AGAAAGCTGGGTCGTGAATGTGCTACGTCAATGAGA | |
| Mo02332 up left For-2 | ATGGCATTGCCATGAGGAGTCTC | Validation of MGG_02332 deletion |
| Mo02332 down right Rev | TATACGCGTCAAACGGTCAATC | |
| attB1 adapter | GGGGACAAGTTTGTACAAAAAAGCAGGCT | For cloning genes by Gateway method |
| attB2 adapter | GGGGACCACTTTGTACAAGAAAGCTGGGT | |
| TrpC Rev | CTAGTGAATGCTCCGTAACACCC | For screening transformants |
| 08454 rFor | CAGTCCGTGCCCAACAGCAA | MGG_08454 QRT forward |
| 08454 rRev | ACGAGTGGCTGCCCGACGTTT | MGG_08454 QRT reverse |
| 10532 rFor | CGGCAAGTACAAAAAGACGACCAGC | MGG_10532 QRT forward |
| 10532 rRev | GTCCATGATGGGCTTGGTGTAGC | MGG_10532 QRT reverse |
| 00401 rFor | GCTCAACAACATGTTCACGGGCA | MGG_00401 QRT forward |
| 00401 rRev | GCAGCGCGACGTCCTTTTCA | MGG_00401 QRT reverse |
| 02332 r2For | GGTCAACATCCGCCAGAAGC | MGG_02332 QRT forward |
| 02332 r2Rev | TCGTCAAACGCACAGTCAAACT | MGG_02332 QRT reverse |
| MGActin F | GGTCTTGAGAGCGGTGGTATCCATG | *Actin* gene (MGG_03982) amplification for QRT |
| MGActin R | TGCCAGGGCAGTGATCTCCTTC | |
| MG40s ribosome For | ATGACCGAGATCATCCAGCGCG | *40S ribosomal protein S3aE* (MGG_06919) amplification for QRT |
| MG40s ribosome Rev | GCTTCAGCAGCTTAACCTTGCGG | |

| | | |
|---|---|---|
| OPR11<br>OPR12 | **TAGAAGCTT**AGCTTGGGCTGCAGGTCGAG<br>**TAGAAGCTT**ATTCCTCGACTCGGTACCCC | PCR amplification for The modified inducible transcription unit |
| OPR13<br>OPR14 | TGCCTGCAGGATATCGTGGAT<br>GCTTGTTTGGGATGTTTTACTCC | PCR amplification for G10-90-XVE cassette. |
| OPR19<br>OPR20 | **AAAAAGCAGGCT**ATGCTTCCCAAGTTCTTCACTCTC<br>**AGAAAGCTGGGT**AGCTCATTGGAAAACCTTGGCCAG | For MGG_08454 cloning |
| OPR21<br>OPR22 | **AAAAAGCAGGCT**AACATGACTAGCAGCGTGTCGTCG<br>**AGAAAGCTGGGT**CAATTAGCCTTGGCTCGTAGCAGG | For MGG_02332 cloning |
| OPR17<br>OPR18 | **AAAAAGCAGGCT**ATTCACAATGAAGTCATTCG<br>**AGAAAGCTGGGT**ATAAAACACATAACCACATAAC | For MGG_10532 cloning |
| 00401 F For<br>OPR24 | **AAAAAGCAGGCT**CCCGGATGCCTAAGCCT<br>**AGAAAGCTGGGT**AAACCTACAACCTGCCACCGCTGC | For MGG_00401 cloning |
| R Actin 1 For<br>R Actin 1 Rev | TGGCATTGCTGACAGGATGAGC<br>CGTCGTACTCAGCCTTGGCAATC | Rice *Actin* gene (XM_015774830) for QRT |
| pTGD For<br>pTGD Rev | CATATGGGAGAGCTCCCAACGC<br>ATATCAGTAGATGCCGACCGC | For construction of pMFKO DONR |
| phh-5 HmB For<br>HSV-tk Rev-2 | ACAGAAGATGATATTGAAGGAGC<br>AAGTTCCTTCCGGTATTGTCTC | For construction of pMFKO DONR |

## References


1 Motteram, J. *et al.* Molecular characterization and functional analysis of *MgNLP*, the sole NPP1 domain-containing protein, from the fungal wheat leaf pathogen *Mycosphaerella graminicola*. *Mol. Plant-Microbe Interact.* **22**, 790-799 (2009).

2 Mattinen, L., Tshuikina, M., Mäe, A. & Pirhonen, M. Identification and characterization of Nip, necrosis-inducing virulence protein of *Erwinia carotovora* subsp. *carotovora*. *Mol. Plant-Microbe Interact.* **17**, 1366-1375 (2004).

3 Garcia, O. *et al.* Characterization of necrosis and ethylene-inducing proteins (NEP) in the basidiomycete *Moniliophthora perniciosa*, the causal agent of witches' broom in *Theobroma cacao*. *Mycol. Res.* **111**, 443-455 (2007).

4 Staats, M., van Baarlen, P., Schouten, A. & van Kan, J. A. Functional analysis of NLP genes from *Botrytis elliptica*. *Mol. Plant Pathol.* **8**, 209-214 (2007).

5 Arenas, Y. C. *et al.* Functional analysis and mode of action of phytotoxic Nep1-like proteins of *Botrytis cinerea*. *Physiol. Mol. Plant Pathol.* **74**, 376-386 (2010).

6 Dallal Bashi, Z., Hegedus, D. D., Buchwaldt, L., Rimmer, S. R. & Borhan, M. H. Expression and regulation of *Sclerotinia sclerotiorum* necrosis and ethylene-inducing peptides (NEPs). *Mol. Plant Pathol.* **11**, 43-53 (2010).

7 Mogga, V. *et al.* *Magnaporthe oryzae* effectors MoHEG13 and MoHEG16 interfere with host infection and MoHEG13 counteracts cell death caused by *Magnaporthe*-NLPs in tobacco. *Plant Cell Rep.*, 1-17 (2016).

8 Santhanam, P. *et al.* Evidence for functional diversification within a fungal NEP1-like protein family. *Mol. Plant-Microbe Interact.* **26**, 278-286 (2013).

9 Cabral, A. *et al.* Nontoxic Nep1-like proteins of the downy mildew pathogen *Hyaloperonospora arabidopsidis*: repression of necrosis-inducing activity by a surface-exposed region. *Mol. Plant-Microbe Interact.* **25**, 697-708 (2012).

10 Dong, S. *et al.* The NLP toxin family in *Phytophthora sojae* includes rapidly evolving groups that lack necrosis-inducing activity. *Mol. Plant-Microbe Interact.* **25**, 896-909 (2012).

11 Feng, B. *et al.* Characterization of necrosis-inducing NLP proteins in *Phytophthora capsici*. *BMC Plant Biol.* **14**, 126 (2014).
